# Supplementary material for: Performance of an extended triage questionnaire to detect suspected cases of Severe Acute Respiratory Syndrome Coronavirus 2 (SARS-CoV-2) infection in obstetric patients: Experience from two large teaching hospitals in Lombardy, Northern Italy
Source: PLoS One. 2020 Sep 15;15(9):e0239173. doi: 10.1371/journal.pone.0239173 (PMC7491723; doi:10.1371/journal.pone.0239173)
Supplement: Attachment — Submitted filename: Response to Reviewers.docx [file pone.0239173.s001.docx]

Dear Referees:

Thank you for the strong positive comments on the first submission of our manuscript. We also appreciate the helpful suggestions as to how we might further improve the paper. Below we respond to each suggestion with details about how we amended the manuscript.

**Response to Referees.**

**Response to Referee 1.**

We appreciate the comments from Referee 1 suggesting our study was of particular interest due to inclusion of minor symptoms in the proposed screening questionnaire. We agree this is extremely important to improve screening efficiency for SARS-CoV-2 infection among obstetric patients at hospital admission.

The Referee asked to rewrite a sentence of the Abstract reporting results of the universal screening period. We addressed this request (Abstract section, Line 36-38).

Referee 1 proposes to perform a ROC curve analysis to assess the questionnaire performance during the study period when a universal SARS-CoV-2 screening approach was implemented.

We thank the reviewer for this comment; however, since the proposed screening questionnaire has a dichotomous result (positive/negative), the most appropriate way of evaluating its accuracy is by calculation of sensitivity, specificity, and positive and negative predictive values in a two-by-two table (as reported in Table 1, Line 182). In turn, a ROC curve analysis with calculation of the area under the curve (AUC) is a more effective measure of assessing a test accuracy when the test generates ordinal or continuous results; sensitivity and specificity can be then computed across all the possible threshold values and vary across the different thresholds [1, 2].

The Referee suggests to specify that a limitation of our study is that RT-PCR on nasopharyngeal swabs for diagnosing SARS-CoV-2 infection was not performed in all women screened by the questionnaire. We appreciate this comment and it has now been addressed in the Discussion section, Line 280-282.

**Response to Referee 2.**

We thank the Referee for the positive comments on our manuscript.

The abstract has now been revised.

Referee 2 suggests to add a section on limitations of our work. This has been done in the Discussion section, Line 280-283.

**References**

1.	Søreide K. Receiver-operating characteristic curve analysis in diagnostic, prognostic and predictive biomarker research. Journal of clinical pathology. 2009;62(1):1-5. Epub 2008/09/27. doi: 10.1136/jcp.2008.061010. PubMed PMID: 18818262.

2.	Hulley S, Cummings S, Browner W, Grady D, Newman T. Designing Clinical Research. Fourth ed: Lippincott Williams & Wilkins, Wolters Kluwer; 2013.
